# Inflammatory cytokine levels and changes during omalizumab treatment in chronic spontaneous urticaria

**DOI:** 10.1007/s00403-024-02966-6

**Published:** 2024-05-25

**Authors:** Selcen Hoşgören-Tekin, İrem Peker Eyüboğlu, Mustafa Akkiprik, Ana Maria Giménez-Arnau, Andaç Salman

**Affiliations:** 1 Department of Dermatology, Marmara University School of Medicine, Istanbul, Turkey; 2 Department of Dermatology, Sultanbeyli State Hospital, Istanbul, Turkey; 3 Department of Medical Biology and Genetics, Marmara University School of Medicine, Istanbul, Turkey; 4 Department of Dermatology, Hospital del Mar and Research Institute of Barcelona, Universitat Pompeu Fabra, Barcelona, Spain; 5 Department of Dermatology Acıbadem Healthcare Group, Acıbadem Mehmet Ali Aydınlar University School of Medicine, Altunizade Hospital, Istanbul, Turkey

**Keywords:** Omalizumab, Treatment, Cytokine, IL-4, IL-5, IL-10, IL-17, IL-33

## Abstract

While several studies have examined the role of T cells and related cytokines in the development of chronic spontaneous urticaria (CSU), there is a limited amount of research focusing on the changes in cytokine levels during omalizumab treatment. The primary objective of this study was to investigate the inflammatory cytokine profile (including IL-4, IL-5, IL-10, IL-13, IL-17, IL-31, IL-33, and TNFα) among CSU patients undergoing to omalizumab treatment. Plasma levels of cytokines were measured using ELISA. Measurements were taken before CSU treatment, at the 3rd and 6th months of omalizumab treatment, and once in the control group. The severity of the patients’ disease was assessed using the weekly Urticaria Activity Score(UAS7), and disease control was evaluated using the Urticaria Control Test(UCT). Thirty-one CSU patients and 56 age- and gender-matched healthy controls were included. Plasma levels of IL-4 and IL-33 were significantly lower in patients with CSU compared to healthy controls (*p* = 0.001; *p* = 0.038, respectively). During omalizumab treatment, IL-4 levels showed a significant increase in the 3rd month compared to baseline (*p* = 0.01), and IL-5 levels significantly decreased in the 6th month compared to both the 3rd month and baseline (6th month vs. baseline; *p* = 0.006, 6th month vs. 3rd month; *p* = 0.001). One potential mechanism of action for omalizumab may involve its regulatory effects on type 2 inflammatory cytokines in CSU patients. This finding partially explains the efficacy of anti-IL-4/13 treatments in chronic spontaneous urticaria. Further investigations on drugs targeting type 2 inflammatory cytokines in CSU are warranted.

## Introduction

Chronic spontaneous urticaria (CSU) is characterized by wheals, angioedema, or both lasting more than 6 weeks spontaneously [1]. In CSU, hives mainly result from mast cell degranulation triggered by abnormal environmental factors rather than intrinsic abnormalities [2, 3]. Various immune cells, including mast cells, basophils, eosinophils, monocytes, neutrophils, and T helper cells (predominantly Th2 subtype), have been detected in urticarial lesions, suggesting their potential role in CSU pathogenesis [4]. Additionally, basopenia and eosinopenia, possibly due to cell migration to wheals during disease activation, are more common in severe CSU cases [5, 6].

Despite numerous studies on the association of various cytokines with CSU severity and activity compared to healthy controls, results have been inconsistent [4]. Additionally, few studies have investigated changes in cytokine levels with omalizumab treatment [4, 7, 8]. Understanding cytokine profile differences between CSU patients and healthy controls, as well as between omalizumab responders and non-responders, could reveal omalizumab’s mechanisms of action, identify new treatment targets, and discover novel treatment response biomarkers. This study aims to assess serum changes in inflammatory cytokine profiles (IL-4, IL-5, IL-10, IL-13, IL-17, IL-31, IL-33, TNFα) of CSU patients during omalizumab treatment at baseline, 3rd, and 6th months. The analyzed cytokines were selected based on previous reports. They were included in the study if a significant difference was found between patients with chronic spontaneous urticaria (CSU) and healthy controls. Additionally, they were chosen from among cytokines that are considered potential therapeutic targets. Secondary objectives include comparing baseline cytokine levels in CSU patients with healthy individuals and exploring cytokine level changes concerning treatment, clinicodemographic characteristics, and omalizumab response.

## Patients and methods

A prospective case-control study was conducted after approval by the institution’s ethics committee (Approval number: 09.2021.10). Patients scheduled for omalizumab treatment due to CSU at a specialized urticaria outpatient clinic between January 2021 and February 2022 were included in this UCARE-certified center [9]. The control group consisted of age- and gender-matched healthy volunteers without any chronic inflammatory disease and history of infection within the previous four weeks. Exclusion criteria included recent anti-inflammatory or omalizumab treatment within the last three months before sample collection, pregnancy, and breastfeeding. The patients in this manuscript have given written informed consent to publication of their case details.

The demographic characteristics, medical history, disease and treatment history of the patients were recorded. Disease severity and disease control were assessed with 7-day Urticaria Activity Score (UAS7) (≤ 6: minimum disease activity; 7–15: mild; 16–27: moderate; ≥28: severe [10]), and Urticaria Control Test (UCT) (≥ 12: well-controlled disease [11, 12]), respectively, at the baseline, 3rd and 6th months of the treatment. The patient group was treated with omalizumab 300 mg every four weeks and second generation H1-antihistamines throughout the study period as per the latest treatment guidelines [1]. The patients were grouped as early or late responders depending on the time of response to omalizumab (response within the first 4 weeks or later) [13].

From all the volunteers participating in the study, blood samples were collected into tubes containing EDTA, three times in the patient group and once in the control group. Blood samples were centrifuged for 15 min at 3000 RPM (revolutions per minute) within 30 min, and the plasma was transferred to eppendorf tubes. Eppendorfs were immediately frozen at -80 °C and stored until the day of the experiment. Cytokine levels in plasma samples were determined with BT-LAB kits by the standard Enzyme-linked immunosorbent assay (ELISA) method. At the same time points, additional venous blood samples were collected to evaluate the complete blood count, total IgE, anti-nuclear antibody (ANA), C-reactive protein (CRP), erythrocyte sedimentation rate (ESR) and thyroid autoantibodies (antithyroglobulin (antiTG), antithyroid peroxidase antibody (antiTPO)) levels in the patient group.

Statistical Package for the Social Sciences (IBM SPSS version 26.0) programme was used for the statistical analysis. The cut-off for statistical significance was determined as *p* < 0.05.

## Results

### Demographics and clinical characteristics

The study included 31 patients with CSU (26 female and 5 male) and 56 age- and gender- matched healthy controls (47 female, 9 male). Table 1 shows the demographic characteristics of the study population. The median duration of the disease was 36 months. In the baseline, 13% of patients showed mild disease, 26% moderate disease, and 61% severe disease according according the UAS7 score. ANA was positive in seven patients, whereas eight patients showed positive antithyroid autoantibody (4 for anti-thyroid peroxidase, 1 for anti-thyroglobulin, 2 for both). Although, only two patients had active thyroid disease. The most frequent comorbidities were chronic inducible urticaria (*n* = 13) and atopic diseases (*n* = 9). The baseline clinical characteristics of the patient group were summarized in Table 1. The analyses were made in 31, 28 and 19 patients in the baseline, 3rd and 6th month of treatment, respectively.

**Table 1:** Clinical and laboratory findings of the patient group

		*n* (%)
Urticaria Phenotype	Urticaria plaque	13 (41,9)
	Urticaria + Angioedema	18 (58,1)
	Angioedema	0 (0)
Chronic Inducible Urticaria	Absent	18 (58,1)
	Present	13 (41,9)
Atopic Comorbidities	Absent	22 (71)
	Present	9 (29)
Autoimmune Comorbidities	Absent	27 (87,1)
	Present	4 (9, 12)
Total IgE level*	< 43 IU/mL	4 (9, 12)
	> 43 IU/mL	27 (87,1)
ANA	Negative	24 (77,4)
	Positive	7 (22, 6)
Antithyroid Autoantibody	Negative	23 (74,2)
	Positive	8 (25, 8)
Eosinopenia	Absent	27 (87,1)
	Present	4 (12, 9)
Basopenia	Absent	27 (87,1)
	Present	4 (12, 9)

* The cut-off value was chosen according to the reference article [19]

### Cytokine levels in patient group vs. healthy controls at the baseline

**Fig. 1:**
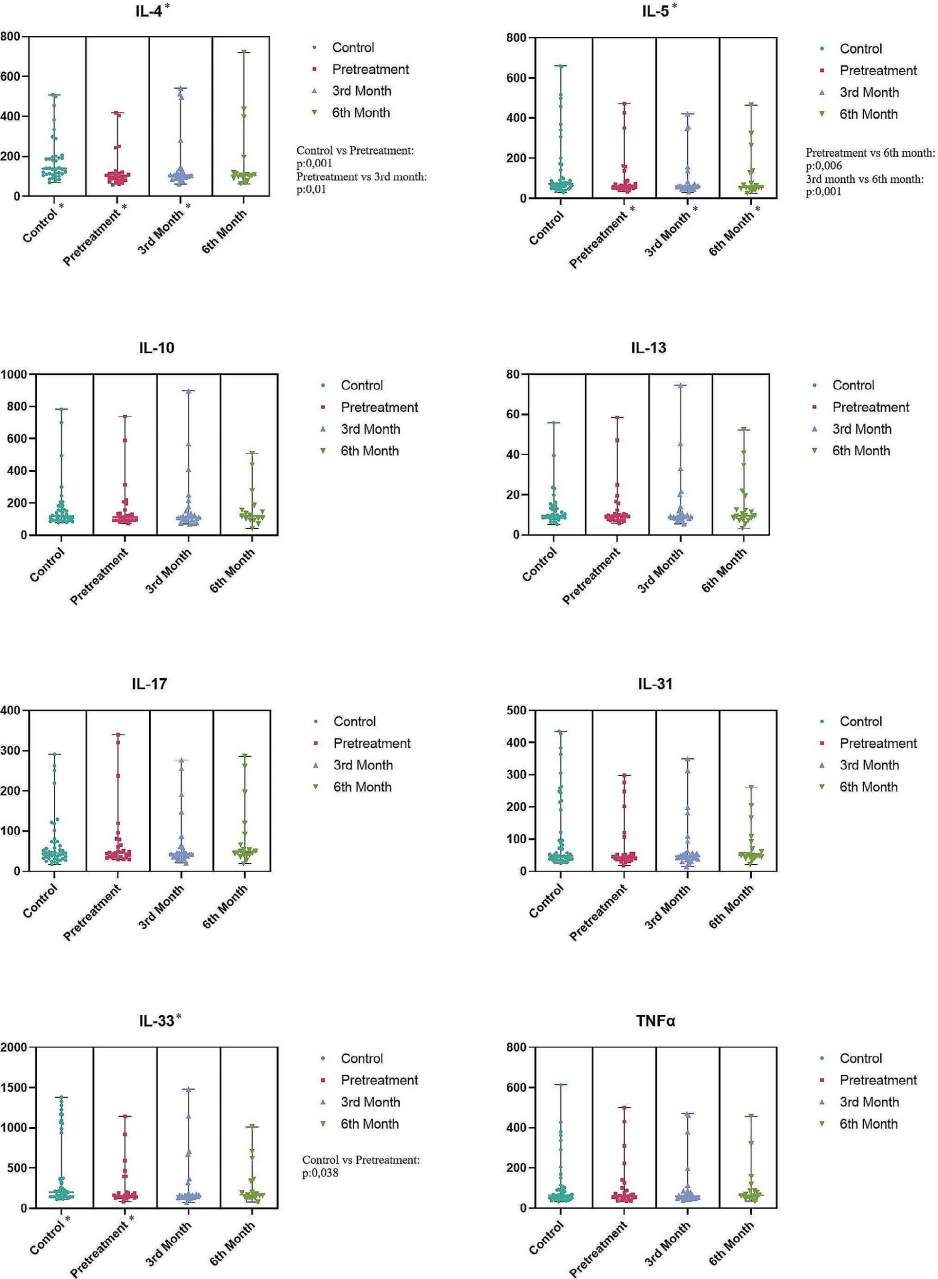
Distribution of cytokine levels in study groups

At the baseline, IL-4 and IL-33 levels were significantly lower in the patient group compared to control group (*p* = 0.001; *p* = 0.038 respectively). There was no significant difference in the levels of IL-5, IL-10, IL-13, IL-17, IL-31, and TNFα between two groups at the baseline. The distribution of cytokine levels in the study groups can be seen in Fig. 1. When the patients were grouped based on the urticaria severity (mild, moderate and severe), IL-10 levels were significantly higher in patients with severe disease activity compared to patients with mild disease activity (*p* = 0.047). IL17 levels was also higher in patients with severe disease activity than in patients with mild disease, albeit not statistically significant (*p* = 0.089).

### Cytokine levels during omalizumab treatment

UAS7-3rd month shows a decrease 20,87 ± 12,11 compared to UAS7-pretreatment; UAS7-6th month shows a decrease of 18,91 ± 11,4 compared to UAS7-pretreatment. When the patients were grouped based on the response to treatment (non-responders (*n* = 2, %6,5) vs. early responders (*n* = 22, %71) vs. late responders (*n* = 7, %22,5)), IL-10 at the baseline and IL-13 at the 3rd month were higher in non-responders compared to early responders, however the difference was not significant (*p* = 0.062).

During the treatment, IL-4 levels increased significantly (3rd month vs. baseline; *p* = 0.01), while IL-5 levels showed a significant decrease (6th month vs. baseline; *p* = 0.006, 6th month vs. 3rd month; *p* = 0.001). Although IL-17 levels tended to decrease with treatment, this did not reach statistical significance (3rd month vs. baseline; *p* = 0.068, 6th month vs. baseline; *p* = 0.059, respectively). The levels of inflammatory cytokines in the study groups and their comparison between groups are summarized in Table 2.

**Table 2:** Cytokine levels in study groups

	Healthy Individuals Median (Q1-Q3)	CSU-Pret. Median (Q1-Q3)	CSU-3rd Month Median (Q1-Q3)	CSU-6th Month Median (Q1-Q3)	Healthy vs. CSU-Pret. *p* value	CSU-Pret. vs. CSU-3rd Month *p* value	CSU-3rd Month vs. CSU-6th Month *p* value	CSU-Pret. vs. CSU-6th Month *p* value
IL-4	138,85 (70,5-508,4)	101,1 (57,8-418,6)	107,35 (62-6-541,8)	106,95 (63,8-720,6)	0,001*	0,010*	0,740	0,569
IL-5	73,1 (30,9-660,3)	61,65 (34,1-471,4)	60,3 (31,2-420,8)	54,9 (25,1-464)	0,187	0,274	0,006*	0,001*
IL-10	119,52 (77,82–782,9)	113,57 (73,24–738,31)	111,59 (70,27–897,36)	124,66 (40,33–654,26)	0,456	0,269	0,758	0,687
IL-13	9,61 (5,22–55,82)	9,3 (5,80 − 58,47)	9,03 (5,57–74,7)	9,63 (3,2–52,28)	0,609	0,136	0,616	0,421
IL-17	45,36 (16,69–291,21)	45,92 (29,37–339,67)	42,58 (20,65–277,17)	49,8 (18,91–285,69)	0,632	0,068	0,184	0,059
IL-31	47,33 (24,99–436,21)	43,69 (18,23–298,67)	46,26 (14,48–350,23)	49,96 (20,61–260,39)	0,153	0,439	0,777	0,573
IL-33	200,53 (108,78-1381,42)	154,89 (85,82-1142,36)	155,29 (70,8-1481,35)	166,93 (77,49-1011,91)	0,038*	0,239	0,811	0,184
TNFα	64,83 (32,84–613,41)	59,57 (35,51–500,46)	57,81 (40,48–470,3)	64,07 (35,49–456,28)	0,716	0,671	0,110	0,586

CSU: Chronic Spontaneous Urticaria, Pret.: Pretreatment

**Fig. 2:**
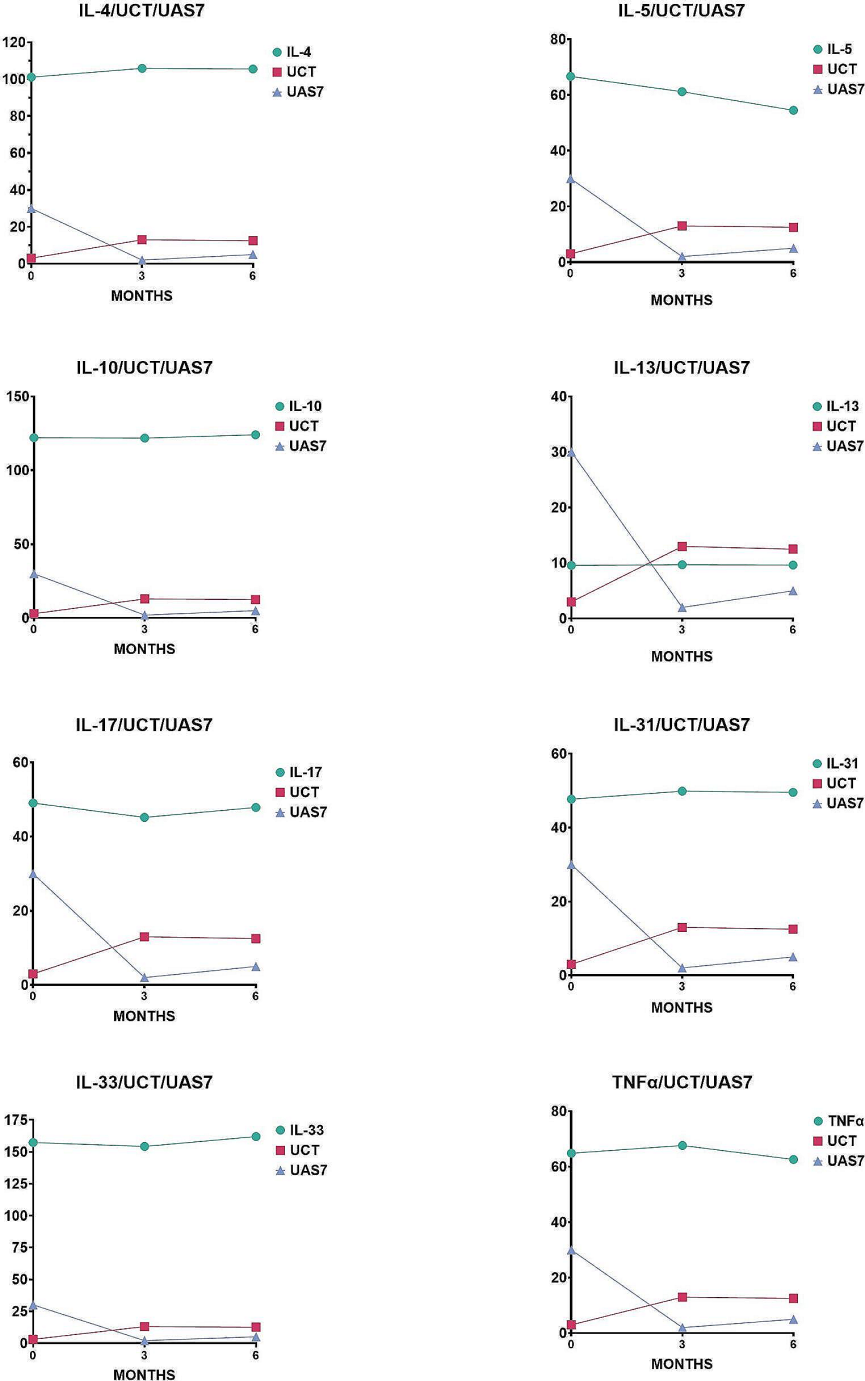
Changes observed in cytokine levels and clinical parameters

When the relationship between changes in cytokine levels and clinical/demographic characteristics was analyzed; the change in IL4 between baseline and 3rd month was negatively correlated with the change in UAS7 scores in the same period (*p* = 0.025, rho= -0.447). IL-4 levels increased at the 3rd month compared to baseline in early responders (*p* = 0.006), on the other hand IL-4 levels decreased in late responders at the 6th month compared to baseline (*p* = 0.027). Changes in cytokine levels and clinical parameters during treatment can be seen in Fig. 2.

In the presence of CIndU, atopy and autoimmunity, there is no decrease in IL5-6th month compared to IL5-3rd month and baseline. While IL-5 levels decreased in late responders between the 3rd and 6th months of treatment (*p* = 0.028); no statistically significant change was found in other groups (*p* > 0.05). When IL5-6th month were evaluated according to IL5-pretreatment, a decrease was observed in both early and late responders (respectively *p* = 0.009; *p* = 0.028), while no statistically significant change was found in nonresponders.

**Fig. 3:**
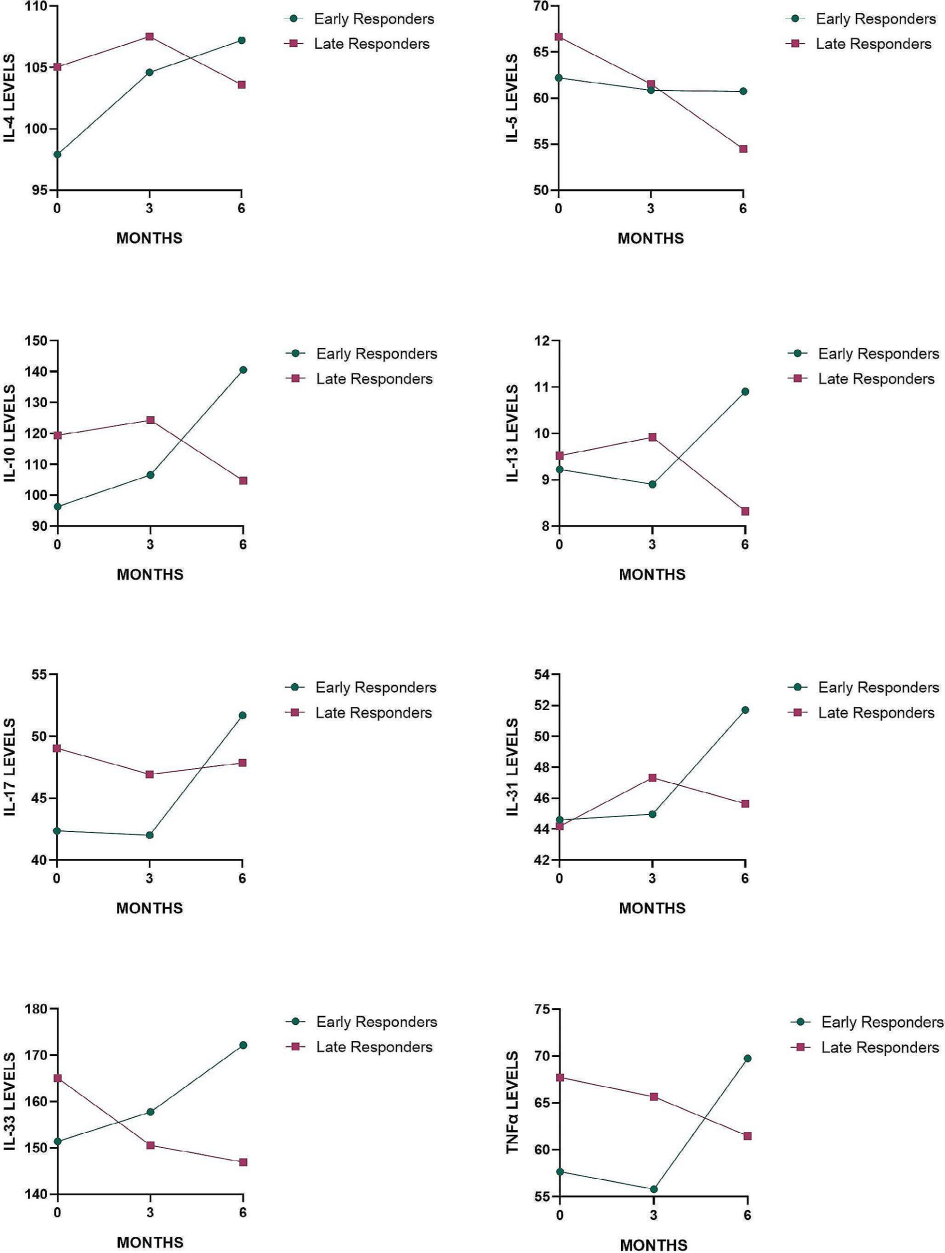
Changes in cytokine levels in early and late responders

IL-17 levels significantly decrease at the 6th month compared to baseline in patients without angioedema and ANA positivity or with more severe disease. The decrease in IL-17 levels at 3rd month was statistically significant in late responders (*p* = 0.028), but no significant change was found in other groups (*p* > 0.05). Inflammatory cytokine levels in early and late responders to treatment and the changes observed in these cytokines during the assessments are summarized in Table 3; Fig. 3. In patients with severe disease, IL-17 decreased significantly at the 6th month when compared to baseline (*p* = 0.022).

**Table 3:** Cytokine levels in early and late responders

	CSU-Slow Resp. Pret. Median (Q1-Q3)	CSU-Slow Resp. 3rd Month Median (Q1-Q3)	CSU-Slow Resp. 6th Month Median (Q1-Q3)	CSU-Slow Resp. Pret. vs. 3rd Month *p* value	CSU-Slow Resp. Pret. vs. 6th Month *p* value	CSU-Slow Resp. 3rd vs. 6th Month *p* value	CSU-Fast Resp. Pret. Median (Q1-Q3)	CSU-Fast Resp. 3rd Month Median (Q1-Q3)	CSU-Fast Resp. 6th Month Median (Q1-Q3)	CSU-Fast Resp. Pret. vs. 3rd Month *p* value	CSU-Fast Resp. Pret. vs. 6th Month *p* value	CSU-Fast Resp. 3rd vs. 6th Month *p* value
IL-4	105,05 (99,9-200,1)	107,5 (90,4-225,9)	103,6 (74,8-199,1)	0,753	0,337	0,027*	97,9 (57,8-405,9)	104,6 (97,9-129,9)	107,2 (93–152,9)	0,006*	0,327	0,779
IL-5	66,65 (53,6-153,2)	61,55 (55,5-151,9)	54,5 (51,1-122,4)	0,602	0,028*	0,028*	62,2 (34,1-471,4)	60,85 (50,9–66,9)	60,75 (52,4-130,2)	0,240	0,009*	0,374
IL-10	119,4 (91,7-177,9)	124,25 (109,7-215,7)	104,71 (93,9-226,5)	0,141	0,471	0,373	96,32 (73,2-738,3)	106,57 (86,2-127,8)	140,57 (112,2-208,2)	0,586	0,799	1,000
IL-13	9,52 (7, 3–14, 1)	9,92 (8, 9–17, 2)	8,32 (7, 2–18, 1)	0,116	0,483	0,274	9,22 (5,8–58,5)	8,98 (7, 9–10, 2)	10,9 (8, 8–20, 1)	0,327	0,386	0,953
IL-17	49,05 (42,6-143,9)	46,93 (38,6-108,7)	47,86 (37,3-114,7)	0,028*	0,075	0,415	42,36 (29,9-320,4)	42,0 (34,3–50,1)	51,69 (38,8–99,1)	0,480	0,323	0,594
IL-31	44,18 (35,7–92,3)	47,33 (34,3–92,6)	45,65 (38,6–86,9)	0,647	0,753	0,641	44,61 (18,2-298,7)	44,96 (39,4–57,0)	51,72 (46,9–96,4)	0,147	0,354	0,859
IL-33	165,07 (132,8-297,5)	150,6 (132,8-312,1)	146,94 (128,9-303,8)	0,463	0,703	0,463	151,43 (85,8-1142,4)	157,74 (142,9-175,8)	172,2 (124,0-343,9)	0,155	0,093	0,678
TNFα	67,7 (53,2-152,9)	65,66 (52,3-159,1)	61,47 (46,2-143,7)	0,607	0,207	0,265	56,6 (35,5-500,5)	55,79 (49,4–72,3)	69,74 (56,9-102,1)	0,396	0,767	0,641

CSU: Chronic Spontaneous Urticaria, Pret.: Pretreatment

## Discussion

CSU is a common condition with wheals, angioedema or both lasting over 6 weeks [14]. Recent studies suggest two CSU endotypes - type 1 and type 2b autoimmunity, with different TH types playing a role in the pathogenesis [4]. Omalizumab is the most effective and safe treatment for CSU, working by preventing IgE binding to FcɛRI, thereby reducing receptor expression [2, 15]. Additionally, it may affect TH cell differentiation through dendritic cells [16].

Conflicting results exist in studies comparing plasma or serum levels of inflammatory cytokines in different types of TH between CSU patients and healthy controls. Few studies have investigated the changes in these cytokines during omalizumab treatment [7, 8]. The present research is crucial for identifying treatment response markers, understanding omalizumab’s mechanisms of action, and potentially discovering new treatments. In our study, 87 volunteers (31 CSU patients and 56 healthy controls) were examined. CSU patients showed lower plasma IL-4 and IL-33 levels than healthy controls. Additionally, in relation to type 2 immunity-related cytokines, IL-4 exhibited an increase during treatment, whereas IL-5 displayed a decrease. Concurrently, elevated plasma levels of IL-10 and IL-13 may indicate a lack of response to the treatment.

IL-4 plays a key role in B cell IgE class switching and differentiation of naive T helper cells to TH2 [17]. It also increases the expression of FcεRI and FcεRII [4, 17]. The increase in IL-4 (IL4-3rd month) compared to baseline (IL4-0) is negatively correlated with the change in UAS7 scores. This increase is observed only in early responders and not in those with prognostic markers for treatment resistance, like eosinopenia [5] or autoantibody positivity [18]. Therefore, an early increase in IL-4 levels during treatment might serve as a marker for omalizumab treatment response.

IL4-3rd month showed an increase compared to IL4-pretreatment and approached levels similar to healthy controls, indicating that omalizumab can regulate cytokine imbalance caused by immune dysregulation in CSU patients. However, no statistically significant change was observed in IL4-6th month compared to IL4-pretreatment. This lack of significance could be attributed to patients’ irregular follow-ups due to the study taking place during the pandemic period, resulting in a decrease in the number of samples available for evaluation at the 6th month.

An alternative theory that could explain the fluctuating change in IL-4 levels is that the initial increase in IL-4 during the early stages of omalizumab treatment might be in response to the sudden decrease in circulating free IgE. This decrease in free IgE could lead to a 2-fold or greater increase in total IgE, which is considered one of the early response indicators to omalizumab [19, 20]. The observed increase in IL4-3rd month among early responders compared to IL4-0, along with the absence of this change in other groups, provides support for this theory.

IL5-6th month showed a decrease compared to both IL5-pretreatment and IL5-3rd month. However, this change did not reach statistical significance in the presence of poor prognostic factors for treatment response, such as autoimmunity and eosinopenia. The decrease in IL-5 levels was observed in responders but not in non-responders, indicating that this reduction in IL-5 levels may be linked to the treatment response and potentially related to omalizumab’s mechanism of action or may be related with the improvement of the disease.

At the 6th month of treatment, the control of the disease was not influenced by age (> 45 years), the presence of angioedema, or the presence of thyroid autoantibodies. However, in the presence of these factors, IL5-6th month decreased compared to IL5-3rd month, whereas no such change was observed in the other groups. This suggests that the decrease in IL5-6th month might be linked to the delayed response of these patients to omalizumab treatment.

Chen et al. conducted a study using ELISA to evaluate serum IL-5 levels in 60 urticaria patients and found a correlation between IL-5 levels and disease severity [21]. In our study, although we didn’t observe a direct relationship, the decrease in IL-5 levels in patients with disease control aligns with the findings of Chen et al.‘s study. Rauber et al. examined IL-5-secreting T cells in 15 CSU patients using the ELIspot test before treatment and at 1-2-3-5th months of treatment. They did not observe a statistically significant change in IL-5 levels and found no correlation with changes in UCT. The smaller sample size and the earlier final evaluation in their study might explain the lack of significant results [8].

The study showed a correlation between the change in IL-33 levels and the change in UAS7 scores (*p* = 0.052, rho: 0.465), suggesting that IL-33 levels tended to decrease in responders to treatment. Although statistical significance might not have been achieved due to the small sample size, these findings support previous publications indicating that IL-33 levels are linked to disease activation [22]. Considering these results, it is possible that IL-33 plays a role in disease severity by stimulating the production of cytokines related to type 2 inflammation.

The late-responder group showed a statistically significant decrease in IL4-6th month compared to IL4-3rd month, along with a decrease or tendency to decrease in other interleukins related to type 2 immune responses (IL-5, IL-33). These findings collectively suggest that the suppression of type 2 inflammation might be one of the late-onset action mechanisms of omalizumab. Additionally, a study conducted with asthma patients also demonstrated decreased IL-5 and IL-13 levels with omalizumab treatment, further supporting this theory [23].

Kay et al. demonstrated an increase in IL-4+/IL-5+/IL-33 + cells in tissue biopsies taken from the lesioned skin of CSU patients compared to those from healthy controls. Furthermore, lesional skin showed an increase in IL-5+/IL-33 + cells compared to non-lesional skin [24]. These findings suggest that TH2 inflammation and related cytokines may play a local rather than a systemic role in the development of hives. In this context, blocking cytokines associated with type 2 inflammation could be effective in the symptomatic treatment of CSU. Indeed, there is growing evidence supporting the effectiveness of biologic agents targeting IL-4, IL-5, and IL-13 in the treatment of CSU [25–28].

IL17-pretreatment levels were found to have a correlation with UAS7 scores at 3 months (*p* = 0.056, rho = 0.365) and tended to be higher in late responders compared to early responders when patients with severe disease were evaluated separately (*p* = 0.091). These results suggest that IL17-pretreatment might serve as a prognostic marker for delayed response, particularly in patients with severe disease at the beginning of the treatment.

It was observed that IL17-pretreatment tended to be higher in patients with severe disease compared to those with mild disease. Additionally, a statistically significant decrease was found in IL17-6th month compared to IL17-pretreatment in patients with severe disease. These findings suggest a potential association between IL-17 levels, disease activation, and treatment response, especially in patients with severe disease. This supports previous publications that have shown a positive correlation between IL-17 levels and disease activity [22, 29, 30].

In the patient group, IL17-pretreatment levels showed a negative correlation with basophil counts (*p* = 0.033, rho= -0.405). Moreover, the change in IL-17 levels between 3 and 6 months positively correlated with the change in CRP levels during the same period (*p* = 0.013, rho = 0.586). Considering that low basophil counts [5] and high CRP levels [31] are associated with disease activation, these findings provide further support for the relationship between IL-17 levels and disease activation.

The mean decrease in IL17-3rd month and IL17-6th month of the patients compared to IL17-pretreatment did not reach statistical significance in the presence of prognostic markers for resistance to omalizumab treatment, such as ANA positivity [18]. However, a statistically significant decrease in IL17-3rd month was observed in the late-responder group compared to IL17-pretreatment, while no significant change was found in the non-responder group. Based on these findings, the lack of decrease in IL17-3rd month compared to IL17-pretreatment could potentially serve as a distinguishing factor between the non-responder and late-responder groups.

The study by Sabag et al. revealed that secukinumab treatment led to an impressive 82% reduction in UAS7 scores at the 90th day in 8 patients with H1 antihistamine and omalizumab resistance [32]. Based on this information, it is possible that the suppression of IL-17 could be an additional action mechanism of omalizumab, particularly in patients with severe disease at the beginning of treatment. Biologic agents targeting this pathway may hold promise for the symptomatic treatment of CSU.

Our study’s strength lies in its evaluation of inflammatory cytokine levels and changes in relation to disease severity, treatment response, and subgroups. While there is a wealth of research on the role of inflammatory cytokines in the pathogenesis of CSU, the literature is limited when it comes to understanding how these cytokines change with treatment. By focusing on these aspects, our study contributes valuable insights to the existing knowledge on the subject.

The primary limitation of our study is the relatively low number of samples available for evaluation at certain measurement points. As a result, some of the observed trends in statistical analyses did not reach the level of statistical significance, possibly due to the limited sample size. Furthermore, another limitation is the inability to conduct tests that support the diagnosis of autoimmune urticaria, such as the autologous serum skin test, basophil activation test, and basophil histamine release test, in the patient group. Consequently, we were unable to analyze subgroups with autoimmune urticaria, which could have provided valuable insights into this specific aspect of the condition. Addressing these limitations in future research may provide a more comprehensive understanding of the topic.

In conclusion, chronic spontaneous urticaria (CSU) is a complex condition with diverse underlying mechanisms involving various inflammatory cytokines and T cell responses. Omalizumab, the most effective treatment for CSU, appears to act by inhibiting IgE binding to FcɛRI and modulating cytokine levels. This prospective case-control study investigated the changes in inflammatory cytokine levels during omalizumab treatment and compared them with healthy controls. The findings indicate potential markers of treatment response and highlight the importance of evaluating cytokine profiles in CSU patients. The study also sheds light on the interplay between cytokines and disease severity, supporting the relevance of type 2 inflammation in CSU pathogenesis and the possible role of drugs targeting this pathway (e.g. Dupilumab) in the treatment of CSU. While this research presents valuable insights, the limitations regarding sample size and the absence of certain tests should be addressed in future multi-center studies to further enhance our understanding of CSU and its treatment. Overall, this study contributes to the growing body of knowledge on CSU and provides a foundation for further exploration of biomarkers of treatment response and novel therapeutic approaches targeting inflammatory cytokines in the management of this challenging condition.

## Data Availability

No datasets were generated or analysed during the current study.
